# Management of human resources for health: implications for health systems efficiency in Kenya

**DOI:** 10.1186/s12913-022-08432-1

**Published:** 2022-08-16

**Authors:** Lizah Nyawira, Benjamin Tsofa, Anita Musiega, Joshua Munywoki, Rebecca G. Njuguna, Kara Hanson, Andrew Mulwa, Sassy Molyneux, Isabel Maina, Charles Normand, Julie Jemutai, Edwine Barasa

**Affiliations:** 1 Health Economics Research Unit, KEMRI-Wellcome Trust Research Programme, Nairobi, Kenya; 2 Health Systems and Research Ethics Department, KEMRI-Wellcome Trust Research Programme, Kilifi, Kenya; 3 Faculty of Public Health and Policy, London School of Hygiene and Tropical Medicine, London, UK; 4 Directorate of Medical Services, preventive and promotive health, Ministry of Health, Nairobi, Kenya; 5 Centre for Tropical Medicine and Global Health, Nuffield Department of Medicine, University of Oxford, Oxford, UK; 6 Health Financing Department, Ministry of Health, Nairobi, Kenya; 7 Centre for Health Policy and Management, Trinity College, the University of Dublin, Dublin, Ireland; 8 Institute of Healthcare Management, Strathmore Business School, Strathmore University, Nairobi, Kenya

**Keywords:** Human resources for Health, Efficiency, Performance, Kenya

## Abstract

**Background:**

Human resources for health consume a substantial share of healthcare resources and determine the efficiency and overall performance of health systems. Under Kenya’s devolved governance, human resources for health are managed by county governments. The aim of this study was to examine how the management of human resources for health influences the efficiency of county health systems in Kenya.

**Methods:**

We conducted a case study using a mixed methods approach in two purposively selected counties in Kenya. We collected data through in-depth interviews (*n* = 46) with national and county level HRH stakeholders, and document and secondary data reviews. We analyzed qualitative data using a thematic approach, and quantitative data using descriptive analysis.

**Results:**

Human resources for health in the selected counties was inadequately financed and there were an insufficient number of health workers, which compromised the input mix of the health system. The scarcity of medical specialists led to inappropriate task shifting where nonspecialized staff took on the roles of specialists with potential undesired impacts on quality of care and health outcomes. The maldistribution of staff in favor of higher-level facilities led to unnecessary referrals to higher level (referral) hospitals and compromised quality of primary healthcare. Delayed salaries, non-harmonized contractual terms and incentives reduced the motivation of health workers. All of these effects are likely to have negative effects on health system efficiency.

**Conclusions:**

Human resources for health management in counties in Kenya could be reformed with likely positive implications for county health system efficiency by increasing the level of funding, resolving funding flow challenges to address the delay of salaries, addressing skill mix challenges, prioritizing the allocation of health workers to lower-level facilities, harmonizing the contractual terms and incentives of health workers, and strengthening monitoring and supervision.

## Background

Universal health coverage (UHC) is a key global health priority and has been included as a Sustainable Development Goal 3 (SDG3) target [1]. UHC aims for quality health services to be accessible to all, when needed, without individuals bearing financial difficulties [2]. In Kenya, UHC is a national priority and is included in the government’s Big Four Agenda [3]. To achieve UHC, additional resources will be required. However, one of the main challenges affecting the Kenyan health system is chronic underfunding [4]. Currently, the government’s spending on health is estimated at 2.3% of the country’s gross domestic product (GDP) against the recommended level of 5% required to achieve UHC [5].

Underfunding of the health sector is addressed by increasing the fiscal space in the health sector [6]. Fiscal space refers to the budgetary room that allows a government to devote resources to specific services or activities without negatively affecting its financial sustainability [7]. There are several ways to increase fiscal space for health; 1) Conducive macroeconomic environment 2) Increased budget allocation to health 3) Earmarking resources 4) Grants and foreign aid and 5) Efficiency gains through improving efficiency within the health sector [8].

Efficiency means optimizing available resources to maximize health system objectives [9]. Given that mobilization of additional resources may not always be feasible, unlocking resources through improving efficiency is considered a potentially viable option [7]. This is because it is estimated that 20–40% of health system spending globally is wasted through inefficiency [10]. Two types of efficiency exist: Allocative efficiency refers to how different resource inputs are combined to produce a mix of different outputs while technical efficiency, is achieving the maximum output with the least cost. Allocative and technical efficiency form the overall efficiency of a health system [9].

A cross-country study assessing the technical efficiency of healthcare systems in 36 African countries using data envelopment analysis (DEA) reported a mean technical efficiency score of 93%, where Kenya’s score was 90% [11]. This study found health worker density to be positively associated with technical efficiency, while the Gini coefficient (a measure of income inequality) to be negatively associated with the technical efficiency of country health systems [11].

To identify ways of improving efficiency and hence realize efficiency gains within the Kenyan health system, the Kenya Efficiency Study sought to examine the technical efficiency of the 47 county health systems and the determinants of technical efficiency within the counties. The first phase of the study aimed at developing a model for conceptualizing efficiency and potential determinants of efficiency in Kenya through an extensive literature review [12] as well as a stakeholder’s engagement forum [13]. In the first phase, the literature review found that national and sub-national health systems that had inadequate numbers of health workers were less efficient [12]. Further, stakeholders in the Kenyan health system identified human resources for health (HRH) as a potential determinant of health system efficiency at the county level [13]. The second phase of the study measured the technical efficiency of the 47 county health systems in Kenya [14]. The study reported a mean technical efficiency of county health systems of 70%, and found that a higher HIV prevalence was associated with lower technical efficiency of county health systems, while higher population density, county absorption of development budgets, and quality of care provided by healthcare facilities were associated with higher county health system efficiency [14]. This phase informed the selection of case study counties for the subsequent phase.

Globally and nationally, HRH has been identified as consuming a significant proportion of health care expenditure hence HRH inefficiencies may significantly affect the overall health system efficiency [10, 15]. There exists a paucity of evidence on how such challenges influence the efficiency of the health system in Kenya. As a third phase of the efficiency study, we aimed to examine the influence of human resources for health on county health system efficiency in Kenya.

## Methods

### Conceptual framework

**Fig. 1:**
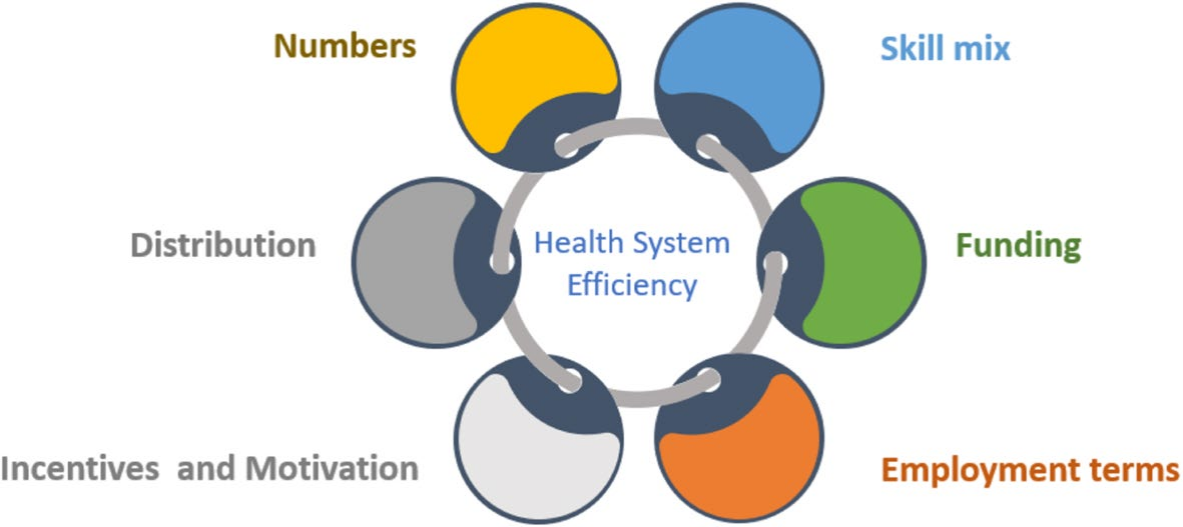
Conceptual framework for analyzing HRH and health system efficiency

We developed a conceptual framework, based on a review of empirical literature on HRH factors that influence health system efficiency. The literature review identified 6 HRH factors that could influence health system efficiency: adequacy of the workforce, skill mix, distribution of the workforce, incentives and motivation of the workforce, contractual arrangements and payment mechanisms of health workers and adequacy of HRH funding (Fig. 1). Adequate human resource numbers overall, their skill mix, and distribution contributes to the effective delivery of quality health services and hence improves health system outputs and outcomes. Inadequate numbers compromise health system functioning with negative impacts for health system outputs and outcomes leading to inefficiency. The adequate funding of the human resource function of the health system ensures that that human resource numbers are sufficient and are motivated. Employment terms affect the costs of the human resource function, their productivity, and motivation. Incentives and motivation affect the productivity of the health workforce, with implications for efficiency. We applied this conceptual framework to our data collection tools, in developing initial themes during the data analysis, and in organizing our study findings.

### Study design

This was a case study design employing a mixed methods approach, with county health systems considered as cases. Two counties were purposively selected based on their efficiency scores from the larger Kenya Efficiency Study (KES) within which this study is nested to include; one high scoring county (County B) and one low scoring county (County A) [14]. These counties have been anonymized to minimize the risk of identifying study respondents. The use of two cases enabled a comparative analysis across different settings which enhanced the richness of our data. Both quantitative and qualitative methods were used to collect data in the two case studies and integrated to support their interpretation.

### Study setting

Data were collected at the national and county levels. Kenya has a devolved system of governance with a national government and 47 semi-autonomous county governments. In relation to health sector roles between the two levels, the national government was assigned HRH training, and broader policy and regulatory roles, while county governments are responsible for health service delivery, priority setting and overall management of health sector service delivery resources including human resources [16]. The public healthcare delivery system is organized into four tiers, namely community, primary care, county referral and national referral. Community health services include all community-based demand creation activities. Primary healthcare includes services provided by public and private maternity homes, health centers and dispensaries. County referral services include first level referral hospitals that are managed by a given county. National referral services comprise of tertiary referral hospitals. HRH management is a fully devolved function; counties have the responsibility for recruitment, remuneration, and overall routine operational management of HRH functions. Table 1 outlines the characteristics of the study counties.

**Table 1:** Characteristics of study counties in 2019

	County A	County B
Population [17]	1,116,436	518,560
Population Density (persons/sq km) [17]	427	54
Urban population [17]	167,200	127,360
Rural population [17]	949,236	391,200
Efficiency score (14)^a^	0.49	0.87

^a^the efficiency scores were computed using data envelopment analysis. The measures represent relative efficiency of county health system and have a range of 0–1

### Study population and data collection

At the national level, data were collected from the ministry of health, development partners, council of governors, healthcare professional associations, health worker unions, and health worker regulatory bodies. In each study county, data were collected at the county administration level, and at the healthcare facility level. Two public healthcare facilities, a hospital (Level 4 or 5) and a primary health facility (Level 2 or 3), where selected in each of the study counties. Individual facilities within the counties were selected through random sampling from the Kenya Master Health Facility List (KHFML). Data were collected using in-depth interviews, and document and secondary data reviews.

### In-depth interviews

Purposive sampling of respondents for the KIIs was carried out based on their roles and experiences in HRH management at national and county levels. A snowballing technique was also applied for identification of additional respondents as this technique has been shown to be useful in reaching hard to reach elites where power and trust are necessary to access them [18].

We included healthcare providers and county officials who had been working in the county for not less than six months to ensure a relatively good understanding of the HRH issues. Data collection stopped at data saturation/the point where no new information was obtained after conducting additional interviews. A total of 46 interviews were conducted. Table 2 outlines the distribution of study respondents across the levels of the health system and study counties.

**Table 2:** Distribution of study respondents across the levels of the health system and study counties

	**County A**	**County B**
Health Facility Managers	5	2
Health Care Providers	3	6
Sub-County Managers	5	3
County Officials	6	4
Union Officials	0	1
Total per County	**19**	**16**
Total both Counties	**35**	
National Level	**11**	
Total Interviews	**46**	

Interviews were conducted using a topic guide whose development was informed by the study’s conceptual framework. Interviews were conducted at private locations in the working stations of the study respondents or an alternative location that the respondents deemed suitable and confidential. All county level interviews were conducted through physical meetings while some national level interviews (*n* = 8) were conducted virtually due to participant preference. Each in-depth interview lasted approximately 45 to 60 min. All interviews were audio recorded using a digital recorder. Peer debriefing was carried out among the researchers after each interview to enhance credibility of the data collected [19]. Reflexivity was adhered to throughout the entire research process to identify any researcher biases and implement strategies to reduce their impact on the data collection, analysis, and interpretation [20].

### Review of documents and secondary data

Collection of secondary data through document reviews entailed collation and analysis of documents and reports containing information on or related to the Kenyan health workforce. Secondary data were obtained from databases and data sources that included materials on HRH and HRH spending as shown in Table 3. Relevant data were extracted from the selected documents using data abstraction guides and transferred to a document review summary form. The collection of data from multiple sources (interviews, documents, information systems) facilitated data triangulation.

**Table 3:** Sources of secondary data

Data Sources	
Information Systems (Ihris, rhris)	County Public Service Human Resources Manual 2013
MoH policy and Strategy documents (Kenya Health Policy 2013–2030, MOH, HRH Norms and Standards Guidelines 2014–2018, The Kenya Health Strategic and Investment Plan, 2014 – 2018)	County Strategic and development plans and reports (Annual Work Plans, County Health Sector Investments Plans, County Integrated Development Plans, Budget reports etc.)
PFM Act 2012; 2015	SRC reports and directives
Collective Bargaining Agreements (CBAs)	Civil Service Code of Regulations

Data collection tools were pre-tested through a pilot exercise to minimize bias and enhance validity of the data collection tools.

### Data management and analysis

#### Qualitative data

Qualitative interview audio files were transcribed into MS Word. The transcripts were then cross-checked against the audio recordings as a quality assurance measure. The transcribed data were then imported into NVIVO 10 software (QSR International, Australia) for coding and to aid with the analysis. Each transcript had a unique identifier comprising of a code, date, and respondent identifier to enhance anonymity and facilitate informed analysis. A thematic approach was employed to provide interpretations and practical recommendations that will be relevant to HRH policymakers. First, a coding framework was developed based on the conceptual framework and preliminary emerging themes. A discussion was then held with all the investigators/researchers to obtain consensus on the final coding framework. All transcripts and documents were then coded using the final coding framework while allowing for the emergence of new themes. Coded data were charted, which entailed summarizing the findings from each transcript based on the various themes and providing illustrative quotes. Data were interpreted by identifying connections between the various themes.

#### Quantitative data

Quantitative data obtained from HRMIS databases (ihris) and county HRH expenditure reports were used to provide descriptive statistics of numbers of health workers, their mix and distribution in the counties as well HRH spending levels in comparison to total county budgets and allocations to various HRH activities. Microsoft Excel was used to analyze quantitative data. Descriptive statistics were used to summarize the key variables as set out in the objectives. Both quantitative and qualitative data were integrated when interpreting the data**.**

## Results

We begin by describing the HRH management processes under devolution in Kenya. We then present the findings in the six thematic areas outlined in the study’s conceptual framework (Fig. 1), namely: HRH funding, number, mix, distribution, contractual arrangements and incentives. These are discussed in turn following an introductory description of HRH processes mapping under county governments. The findings per county are also summarized in Table 4.

**Table 4:** Summary of findings in the case study counties

	**County A**	**County B**
HRH Funding	• Inadequate HRH funding • Delays in salaries reported	• Inadequate HRH funding • No delays in salaries reported
HRH Numbers	• Staffing shortages experienced for all cadres except general clinical officers	• Staffing shortages experienced for all cadres except general clinical officers
HRH Skill-Mix	• Inadequate skill-mix with specialist shortages	• Inadequate skill-mix with specialist shortages
HRH Distribution	• Mal-distribution of workers skewed towards hospitals	• Maldistribution health of workers towards hospitals
HRH Contractual arrangements	• Incoming health workers employed on Permanent and Pensionable (P & P) basis	• Incoming health workers employed on fixed term contracts
HRH Incentives and motivation	• Inter-cadre disparities in training opportunities reported • Risk allowance for non-service delivery staff eg HR managers, accountants etc • Absenteeism was reported	• Rural staff had differential opportunities for transfers • Risk allowance for non-service delivery staff eg HR managers, accountants etc • Absenteeism was reported

### HRH processes mapping under devolution in Kenya

Following the implementation of the constitution of Kenya 2010, health service delivery (and subsequently HRH) became a fully devolved function in Kenya [21]. County governments were charged with the responsibility of undertaking all the routine HRH management processes including recruitment, deployment, in-service training and payments of salaries [22, 23]. Under the devolved governments, the county department of health (CDoH) is responsible for planning its HRH requirements within the budget provided by the county treasury [23]. The County Public Service Board (CPSB) has the overall responsibility for the recruitment of all county government employees, including health workers [21]. Once recruited, the department of health has the mandate to deploy the health workers to various stations based on need as well as manage the health workers including payment of salaries, performance appraisal, promotions and training of the health workers [24].The national government, through the MoH, retains responsibility for pre-service training and development of broader policy guidelines.

### Funding for human resources for health

#### HRH funding in the study counties was inadequate

Study respondents felt that this was because of the overall inadequate levels of financing for health in the counties. Additionally, late budgeting for HRH needs had affected the amounts allocated for hiring, training and promoting health workers in County A. Inadequate HRH funding was seen to limit the ability of the counties to recruit an adequate number of health workers to meet the country’s staffing requirements as per the Kenya health worker staffing norms [25]. This was thought to constrain the county health system’s ability to function and hence negative implications for health system efficiency.

“We are constrained by the limited budget lines. If we could have adequate funds, then we would be able to employ according to the requirements of the norms and meet our objectives in a smooth manner.” (HR Manager, County A)

“In the previous budgetary cycles, the health department would not factor in sufficient money for HRH recruitment and promotions. So, when it suddenly arose, we did not know how to help them because once it is not in the budget, it cannot be done. We have since sensitized them and now I think it is being done correctly.” (Senior Economist—Finance Department, County A)

Inadequate funds also affected the ability of counties to invest in in-service training of health workers. Respondents felt that this reduced the ability of counties to retain health workers, potentially worsening the adequacy of numbers of human resources in the county and increasing employment costs because of need to regularly recruit to replace staff with potential negative implications for health system efficiency. It also constrained capacity development for the health workforce.

“We don't sponsor their tuition, but we offer them paid study leave. It saves us costs on one hand but again, it might be the reason the attrition number is high. The staff who pay for [their own training costs] themselves are not bonded like those who might have had their tuition paid. So, when they finish training it is easy for them to move to other areas and we have to recruit new health workers” (Medical Superintendent, County A)

#### Health workers in county A experienced delay in payment of salaries

Respondents attributed delays in salaries to delays in the disbursement of funds from the national government to the county government, and the prioritization of funding by counties to capital projects over staff salaries. Delays in salary payments were a major source of health worker demotivation and frequent health workers’ strikes in county A with likely negative impacts on health system efficiency.

“Disbursement delays are an issue out of our own hands at the county level. The delays are as a result of delays from the national treasury. Once the funds are available every person gets his/her salary” (Senior Economist, County A)

“In some counties, disbursements may have been done but they move this money to build roads. Then they wait for other development projects funds so that they can pay salaries. The workers are paid late and their motivation is affected. It also leads to frequent strikes.” (Professional body 2 Respondent)

The situation was different in county B, which mitigated the delayed fund disbursements from the national government by negotiating credit facilities with local banks and prioritizing staff salaries in the allocation of locally generated revenues.

“In our county, we have negotiated with a local leading bank. They pay our health workers’ salaries promptly. We are able to pay our workers their net salaries on time and they are able to continue discharging their duties with minimal disruptions.” (Payroll Manager, County B)

### HRH Number, Skill-Mix, and Distribution

#### HRH staffing shortages were reported in both study counties

This was supported by document and secondary data review which showed that there were significant disparities in the availability of health workers relative to the Kenya health sector staffing norms [25]. Table 5 outlines selected tracer cadres in 2020/21 relative to the recommended staffing norms for both counties [26, 27]. Tracer cadres show that county A had achieved 19% of staffing relative to the staffing norms and standards while County B had achieved 35%. Shortages of health workers were attributed to the inadequacy of HRH funding. Shortages in HRH constrained the system’s capacity to function with likely negative implications for health system performance generally and efficiency in general.

“If you pick one health indicator such as skilled birth attendance and look at your catchment populace, you'll realize that there's a percentage of women whose deliveries are not being conducted by a skilled birth attendant. This is mostly due to having only one or few nurses at facilities since the health center cannot operate 24 h and on weekends.” (Medical Superintendent, County B)

**Table 5:** Comparison of cadres and staffing norms by level of care in County A and B 2020/21 Annual Work Plans (AWPs)

	**Staff Cadre**	**County A**			**County B**		
		**Employed**	**Required according to staffing norms**	**% as per norms met**	**Employed**	**Norms & Standards**	**% as per norms met**
**1**	Medical officers	32	200	**16.0**	63	76	**82.9**
**2**	Medical specialists	7	-	**-**	21	-	**-**
**3**	Clinical officers (specialists)	24	160	**15.0**	10	62	**16.1**
**4**	Clinical Officers (general)	155	130	**119.23**	64	50	**128**
**5**	Nurses (registered)	499	860	**58.0**	188	340	**55.3**
**6**	Nurses (Enrolled)	149	1640	**9.0**	231	656	**35.2**
**7**	Total	950	4303	**22.1**	805	1683	**47.8**
**8**	Health Workforce Density	8.5	44.5	**19.1**	15.52	44.5	**34.9**

However, from our document review, staffing excesses for general clinical officers were noted in both counties relative to the recommended staffing norms and standards for the county (Table 5). This was attributed to the fact that donor programs boosted the numbers of certain cadres of workers such as clinical officers and could therefore result in higher numbers of these health workers.

“Donor programs have provided resources to hire additional health workers such as clinical officers and nurses which have boosted our HRH numbers and relieved our overall HRH shortages” (Medical superintendent, County B)

### Health workers skill-mix

#### Case study counties had inadequate skill mix characterized by scarcity of specialists

Specialist health workers such as medical specialists and intensive care unit (ICU) nurses were reported to be scarce in both counties. Specialist shortages constrained the capacity of counties to deliver services to people that needed specialized health services, with potential implications for health outcomes and hence health system efficiency.

“One of our biggest health concerns in this area is non- communicable diseases. We have very many patients with hypertension and diabetes. Even asthma and COPD due to perennial use of firewood. But we don’t have enough doctors to deal with the cases” (Medical Superintendent, County B)

Further, specialist shortages had resulted in inappropriate task shifting whereby nurses and medical officers performed specialist duties in the absence of specialists. This compromised the quality of care with likely negative impacts on health outcomes and hence efficiency.

“The citizens may not get quality services. For example, my boss was complaining that she had been advised to wear plaster for a whole week, only to later find a specialist who found out that she had been mis-managed because she had been attended at our general hospital by someone who is not a specialist” (Payroll Manager, County B)

“We require the specialists here because sometimes we are forced to risk. For example, one may refer a pregnant mother who has a previous scar, but she may refuse to go and just come at night and the nurse is forced to risk. The nurse attends to them while praying that nothing goes wrong.” (Nurse, County B)

County A reported two ways in which skill-mix imbalances had been addressed within the county to improve efficiency; i) On-job training of nurses and clinical officers and nurses ii) sharing of specialists across facilities and with neighboring counties. These measures were reported to improve county health system efficiency by reducing the costs of recruiting and maintaining specialists in the county or within one facility.

“It is cheaper for this county when we engage external specialists from other counties because the image is still taken at the same cost and the county does not pay this specialist a salary. For example, from the amount the patients pay, we have an agreed amount that we pay to the radiologist who is interpret the image. It is quite economical.” (Deputy Medical Superintendent, County A)

“Occasionally, the experts move to where the staff are and mentor them on what they are meant to do. This helps us fill the gap that is there in terms of skill mix. For example, we had just one renal nurse at the time and the rest of the nurses who joined the team were trained on the job.” (Deputy Medical Superintendent, County A)

#### Both study counties were characterized by the maldistribution of health workers

Distribution of health workers was perceived to be skewed towards higher level care compared to primary healthcare (PHC) facilities. Respondents felt that the skewed distribution of health workers affected the quality of care at primary health care. It also resulted in unnecessary referrals to the higher-level facilities, increased the workload for the health workers in referral facilities, and increased the cost of care since care in hospitals was more expensive. All these had potential negative implications for health systems efficiency.

“Shortages of staff in the lower level facilities affect our performance because of a lot of unnecessary referrals to the hospitals. Most cases that we feel ought to have been handled at that level end up here. This delays interventions for the patients.” (Deputy Medical Superintendent, County A)

“We also have some gaps in documentation due to the rush to attend to the other patient when you have only one staff in a facility.” (Nursing Officer L3, County A)

### Health worker employment arrangements

One of the guiding principles outlined in the devolution guidelines for HRH 2015 is a commitment by counties to harmonize and standardize contracting mechanisms for health workers [24]. Our study found several types of contracting practices for health workers at the county level: those for i) permanent and pensionable staff (P and P) ii) fixed-term contract staff iii) Locum contract staff.

#### Varied contractual arrangements within similar skill-sets performing similar duties were reported in both counties

County A employed all its health workers on P and P basis except for casuals while County B hired all incoming health workers on fixed-term contract terms. Other health workers within the two counties who were under fixed-term contract terms included staff seconded from MOH, donor or partner-staff and casual workers. In addition to these modes of contract, our study found a more recent practice of hiring health workers on short-term locum basis in both study counties. The different contractual arrangements were seen to have different incentives for the performance of the health workers. While some respondents felt that fixed-term contract terms incentivized employees to perform better to secure contract renewal, others felt P and P terms provided more security to the workers who were then able to perform optimally.

“As a health worker on contract basis myself, we achieve more because we are given targets. We have to achieve those targets to remain in that contract which is a positive thing because our colleagues who are on permanent terms are a little bit relaxed in their outputs.” (Health Worker, County B)

“P an P employees perform better. Contract employees especially towards the end of the contract, spend most of their time trying to secure their future by looking for other jobs. They are not settled at work during this time and their motivation is low.” (Medical Superintendent, County A)

In county B, it was reported that workers on permanent contracts could access commercial loans from banks while fixed-term contract staff could not. Pension, long-term training opportunities and comprehensive medical covers were also highlighted as important differences between P and P staff and the fixed-term contract staff. This contributed to a sense that staff with permanent contracts had advantages over those on fixed-term contracts, contributing to demotivation of the latter, with potential negative implications for quality.

“Those on permanent and pensionable terms have more benefits such as access to long-term mortgages and car loans, pension, long-term training opportunities and comprehensive medical covers.” (Union Official)

Further, inadequate compensation for health workers on fixed-term contract terms was reported to affect their motivation. This is because short term contracts were viewed as riskier and would hence require higher levels of compensation. It was also reported that there were no mechanisms in place to absorb donor/partner contracted staff into the county health system after their terms expired. Short term contracts were thought to increase employment costs given the need to regularly recruit staff, and to compromise health workforce capacity through the loss of skilled staff.

“I don’t think there is anything particularly wrong with contracts, they only need to be fair compensation to the worker…The counties should also look at sustainability and continuity. If you hire an obstetrician for 3 years, they disappear then you must train another one and hire them for 3 years, where is the continuity of services in that?” (Professional body 2)

“The problem is that donor/partner programs hire and train staff who are later not absorbed into the system after the project ends because there are no direct policy guidelines for that. When the board hires other people other than these people, human capital is lost.” (Director Public Health, County A)

#### The informal part-time working arrangements for specialists, who also engaged in dual practice was thought to be inefficient

Specialized staff did not work fulltime but rather only when there were cases that needed specialist attention. This was partly because they had dual practice, allocating some of their time to their private clinical practice. The specialists were however paid fixed salaries that assumed they worked full-time in their public facility duty stations. Respondents thus felt that payments for specialists was not aligned with their level of effort and time input.

“Doctors work very few hours in the hospital and then they leave for other commitments. I do not think that they are giving us the maximum value, because it is very difficult to find a doctor working for 8 h in the government facility, yet they are paid a lot.” (Hospital Administrator, County A)

### Health Worker Incentive and motivation

According to the Kenya HRH strategy 2014–2018 [22], counties have the mandate to develop an incentive policy for attraction and retention of their health workers**.** This includes creating an environment where health facilities located in hardship areas are protected from staff shortages by the use of various incentives. The range of financial and non-financial incentives available for health workers is outlined in Table 6.

**Table 6:** Financial and non-financial incentives for health workers

Financial incentives	Non-Financial incentives
A mid-range entry level basic pay in hardship areas that is higher than the normal areas for new entrants to the service with bonding to ensure it serves the attraction and retention expectation	Provision of comprehensive health care services for health workforce and immediate family
A higher house allowance than the normal working areas if housing is not provided	Opportunities for continuous professional development, such as a prioritized post-graduate training after serving a certain number of years
A hardship allowance paid to members of staff who are stationed in the designated hardship areas	Improved human resources management (HRM) including; reduced workloads, supportive supervision, decentralization of human resources activities, deployment on areas of choice or having fixed term in hardship areas, clear roles and responsibilities within their job description and performance appraisals
A higher non-practicing allowance (to compensate health workers for not engaging in dual practice) paid to doctor and dentists who are not practicing than normal areas	Access to house, education or car loans at lower negotiated market rates (for highly skilled public sector workers)
An additional responsibility/duty allowance paid to officers who are required to handle tasks beyond their job descriptions, such as acting as head of a department, nurses who act as professional counselors in facilities and members of sub County Health Management Teams (SCHMTs)	Establishment of social amenities within vicinity of the facility such as staff canteen, gym facility, recreational centers

*Source*: Devolved Policy Guidelines on Human Resources for Health in Kenya, 2015

#### Study respondents reported disparities in incentive structures across counties, cadres, contract type, levels of care, and geographical regions

First, inter-county disparities in incentive structures led to health workers in one county obtaining favorable incentives while their counterparts in another county had less or no incentives. This is because different counties had the prerogative to decide their own incentive structures such as promotions, car loans and mortgages. Inter-county disparities in incentives for health workers resulted in demotivation among health workers and sub-optimal performance with likely negative impacts on health system efficiency. It also resulted in the loss of health workers to counties with better incentive packages. The loss of human capital was costly to the counties due to the costs involved in recruiting and training new health workers.

“Some counties are offering car loans and mortgages for their health workers as an incentive in addition to other incentives which has a demotivation aspect to other workers because some counties do not implement such.” (Union Official)

“A clinical officer like me will be in one job group in one county and a higher job group in another county. There's a lot of discrepancies that have been created. These people compare notes and it creates a lot of disharmony, unrest and attrition.” (Union Official 2)

Second, inter-cadre disparities in training opportunities were highlighted in County A. It was reported that training opportunities were preferentially given to doctors and nurses while the other cadres were largely left out. It was also reported that those in senior management positions at the county level were sponsored for short courses while the other staff were not sponsored for the same. Additionally, non-service delivery staff such as accountants, human resource managers and administrators were often not considered for training opportunities. Lastly, differential training opportunities across levels of care were reported. Those in PHC facilities were seen to have more training opportunities than those in higher level facilities. This was attributed to the fact that most donor activities were concentrated at PHC facilities and hence provided more training opportunities for those in the PHC facilities. Differential opportunities in training of the health staff were reported to influence the knowledge level and motivation of other health workers negatively in county A with likely negative impacts on health system efficiency.

“It is difficult to understand how they select the people to go for training because mostly the county pays for the doctors and sometimes nurses without concentrating on the other cadres which ends up demotivating the rest” (Hospital Administrator, County A).

“One example is the short courses at Kenya School of Government. Sponsoring should not only be limited to the CHMT members, but it should be across all staff based on job groups and the requirements so that the knowledge gap is not too wide across the staff.” (SCMOH, County A)

Third, respondents reported disparities in risk allowances in favor of staff that provided direct health services. Non-service delivery staff such as accountants and administrators did not receive risk allowance. This resulted in demotivation with likely negative implications for health system efficiency.

“A serious challenge we face is that non-health service workers such as accountants, human resource officers, administrative officers are not paid risk allowance, yet they interact widely with patients and the health workers. It is risky for them too. Training and development for these staff is never taken seriously too yet they are also part of the system.” (HR Manager, County A)

Finally, it was reported that rural health workers were often overlooked in transfer opportunities compared to their urban counterparts. This resulted in demotivated health workers and sub-optimal performance within the rural areas.

“We feel like we are often overlooked here in the rural facilities. It is difficult to get a transfer. Some staffs have worked in this rural area for many years, but they don’t get transfers. This has demotivated some staff.” (Health Worker, County B)

#### Absenteeism by health workers was also reported

Absenteeism took the form of illegitimate sick-offs, unexplained absence from work by health workers, and informal arrangements by health workers to work in shifts. This was corroborated by the Kenya service delivery indicator survey (2018) that reported an absenteeism rate of 47% in county A and 45% in county B [28]. Respondents also reported cases of complete abscondment of health workers from duty for prolonged periods (these were referred to as ‘ghost workers’).

“Somebody may be absent because this person is sick, this person is, is indisposed…So there is a mechanism under which some of these cases are heard and determined. In other facilities, like for example, if you go to a dispensary, maybe you have three officers or two, they could make their own local arrangement so that there's always one nurse on duty, the other one is always absent. And yet the principal is both of them must be on duty.” (County Manager 4, County A)

One of the key reasons attributed to absenteeism was weak accountability mechanisms that made it difficult to address the problem.

“Occasionally, you get one or two members of staff in patterns of absenteeism. Part of the reason why they disappear is because we, as the supervisors are not near that facility. When you get funding for supervisory visits, you find this staff is not there. And maybe this might just be your first time to find him absent. It’s only after you have visited severally that you realize, oh kumbe (so) there is a pattern, he’s never there.” (County Manager 1, County B)

It was reported that the presence of ‘ghost workers’ hindered the recruitment of adequate numbers of health workers by counties. This was because ghost workers were counted as part of the numbers of the total stock of county staff, contributing the attainment of staff count ceilings, and thus reducing the allowable number of additional staff that could be recruited by counties.

“…there are those ones as I told you, the ones I called ghost workers. You see that is high level because people are put on payroll. For example, they say there are 1000 nurses but the ones we manage are 800. So already 200, you have never seen them… It is only when you have access to the payroll that you realise that there are more people than you thought there are” (County Manager 2, County 1)

Absenteeism was thought to negatively impact on healthcare service delivery. This led to an inadequate number of staff to handle the demand for healthcare services.

“Generally, clients will suffer (due to absenteeism), and it might lead to increased mortality in our facilities. You find that probably when there's an emergency somewhere, and the health care worker is not there. You see that client might succumb.” (County Manager 9, County 1)

“Of course, now, when you’re absent it means the service delivery is affected wherever you are. So, if you're a doctor, your clients will not get the services, if you’re a nurse the same. The children maybe they came for immunization they will not find you. So it ends up creating a situation whereby you are spending but the outputs are not coming forth. Just spending but the outputs expected” (County Manager 1, County 2)

Absenteeism was thought to contribute to the demotivation of health workers that were present at work.

“Some of the health workers they have godfathers in the counties. And they have that feeling that the immediate supervisors or any other supervisor, doesn’t have power over them… and sometimes it(absenteeism) becomes infectious to the other personnel. If you don't take action against one who’s doing that (absenteeism) then it will bring another staff becoming absent from his work.” (County Manager 1, County 2)

## Discussion

This study examined human resource management practices in county health systems in Kenya and their implications for health system efficiency. We found that human resource management at the county level was characterized by inadequate funding, delayed salaries, inadequate HRH numbers, improper skill-mix, skewed distribution of health workers, variable contractual mechanisms and disparities in incentive structures for health workers. These HRH management practices could potentially influence the efficiency of county health systems in several ways.

First, inadequate recruitment of health workers and limited resources for in-service training, occasioned by inadequate funding and caps on staff recruitments, is likely to compromise the optimal mix and quality (health worker knowledge and skills) of health system inputs with negative impacts on health system outcomes and hence efficiency. Limited access to health care and poorer outcomes have been associated with understaffing, resulting in inefficient health systems [29]. For example, in a cross-country study a higher health workforce density was associated with decreases in maternal and infant mortality rates [30, 31], and decrease the total burden of disease [32]. Many studies have highlighted the difficulty by health systems in achieving optimal HRH numbers [29, 33–37]. While donor support was highlighted in Kenya as a potential path to increasing funding to hire and train health workers, experiences from elsewhere shows that the reliance on external funding for HRH scale-up needs to be carefully considered in view of sustainability [38].

Second, increased turnover is likely to increase health system employment costs, and hence introduce inefficiencies in the health system. Increased staff turnover was the result of reduced staff motivation because of limited county resources and hence opportunities for training, delays in salaries, and frustrations over differences in contract terms and incentives among county health workers. Reduced staff motivation also led to health worker strikes and is likely is to result in reduced staff productivity, reducing the efficiency of county health systems. Health worker motivation has been shown to impact negatively on health worker productivity and hence efficiency in other settings. Determinants of health worker motivation span financial and non-financial and include some of the factors that were highlighted by our studies such as timeliness in payment of salaries [13], and perceptions about preferential incentives for different cadres of health workers [39].

Third, the scarcity of medical specialists and use of non-specialist staff to provide specialist health services constrains the capacity of counties to provide continuity of care and specialist services, and is likely to compromise quality of care, outcomes and hence the efficiency of the health system. This resonates with several other studies that have reported shortage of specialists in the counties [40]. Our findings corroborate findings from another study in Kenya that found that young and newly qualified health workers had to work without supervision due to attrition of specialists from the system within 5 years of service [40]. Studies across a range of countries have shown that skill flexibility (role substitution and delegation) can be beneficial to the efficiency of a health system [41–45]. However, skill-development through role enhancement is crucial to provide the pre-requisite skills for higher level responsibilities of staff that take on new roles [44].

Fourth, the informal part-time working arrangements for medical specialists who were reported to engage in dual practice is likely to contribute to inefficiency by misaligning health worker effort with compensation. Payment methods for healthcare workers has been shown to affect costs, quality of care, and efficiency of health systems [46–49].

Lastly, the inadequate numbers of health workers at the primary healthcare level (health centers and dispensaries), occasioned by the distribution of staff in favor of higher-level health facilities (referral hospitals), compromised the quality of care at the PHC level and led to unnecessary referrals to referral hospitals, likely introducing inefficiencies to the system. Other studies have reported mal-distribution of health workers as a cause of health system inefficiency in many low-middle income countries (LMICs) [40, 50, 51]. For example, a systematic review of literature highlighted that mal-distribution can lead to underuse of skilled personnel while increasing the total cost of health care system [51].

We did not find systematic differences in human resource management practices between the county that was ranked as efficient and the one that was ranked as inefficient by the quantitative efficiency analysis. This could be because variation in human resource management practices between the counties are in terms of intensity rather than occurrence and hence are difficult to tease out using a qualitative approach. It could also be because the counties that were ranked as efficient by the quantitative analysis by being on the efficiency frontier are inefficient in absolute terms, even though they are relatively more efficient than the counties that at a distance from the frontier.

Our study has several limitations. First, the results from two counties out of 47 counties may limit the generalizability of the study findings to the entire country. However, given that HRH operations do not differ widely among the counties, the results obtained in the study could provide useful insights to other counties. Second, the study did not collect data from patients, local political leaders, and the private sector, all of whom could provide valuable additional insights. Finally, HRH data is fragmented and incomplete within the various HRH databases. Sourcing of the data from different sources with varying data could present inaccuracies. We found discrepancies in HRH data contained within the county AWPs, the national database (ihris) and county records. In such cases, we used the most complete data and sought validation from the county HRH Managers. We also could not find good quality quantitative data to triangulate all our qualitative findings.

These limitations notwithstanding, the study highlights several potential policy levers for improving HRH management at the county level that could improve county health system efficiency. First, county governments should assess and align their budget allocations to their human resource function with updated assessments of county human resource capacity needs in terms of numbers of staff and required capacity development. This will need to be done while appreciating the fiscal constraints that counties face. Options for optimizing resources use in HRH such as task shifting may close gaps in health workers quantities cost-effectively [52]. Second, counties should review and seek to resolve funding flow challenges that contribute to delays of salary payments to health workers. This problem is partly a broader public finance management (PFM) challenge characterized by delays in disbursement of funds to counties and from county treasuries to county departments of health and will hence require the strengthening of PFM implementation processes [53]. However, at the county level, it could also be resolved prioritizing the payment of health worker salaries before making other payments. Third, counties should optimize the skill mix of health workforce, informed by an assessment of skill needs and gaps at different levels of care based on locally developed (country level), practical staffing norms. Specifically, counties should assess their needs for specialist health worker and seek to fill this gap. A cost-effective approach for countries would include developing a mechanism for sharing specialists across counties rather than having each county seek to recruit their own specialist health workers. This will require the strengthening of inter-county coordinating mechanism by developing and implementing health workforce sharing mechanisms. Fourth, counties should harmonize contractual terms and incentives within the county, and across counties. While the jury is still out on what contractual terms will be most ideal, harmonizing terms for staff with the same skills and roles and developing clear guidelines for the application of specific contractual terms should be explored. Fifth, counties should explore output-based payment methods for medical specialists such as capitation or case-based methods or blended methods that combine two mechanisms to align their payments and outputs and hence enhance efficiency. Lastly, and perhaps overarching, the governance and institutional capacity for HRH at the county level should be strengthened. This includes strengthening HRH county level HRH coordination, and harmonized structures, policies and process for HRH management (planning, recruitment, deployment, compensation, information systems etc.).

## Conclusion

This study has identified human resource management practices at the county level in Kenya that have implications for health system functions, outcomes, and efficiency. These impacts are mediated through effects on optimal health system inputs, quality of health system inputs and outputs, healthcare costs, and health worker motivation. The study identifies potential policy levers that county health systems in Kenya could target to improve human resource management and health system efficiency. While these findings and conclusions are based on data from Kenya, they are potentially relevant in other LMIC settings with similar contexts. Further research is needed to examine optimal contractual, remuneration, and incentive structures in Kenya and similar LMIC settings.

## Data Availability

The datasets generated and/or analysed during the current study are not publicly available due to participant confidentiality but are available from the corresponding author [EB] on reasonable request.

## Abbreviations

AWP: Annual Work Plan
CBA: Collective Bargaining Agreement
CDOH: County Department of Health
COPD: Chronic Obstructive Pulmonary Disease
CPSB: County Public Service Board
GDP: Gross Domestic Product
HRH: Human Resources for Health
HRM: Human Resource management
ICU: Intensive Care Unit
irhis: Integrated Human Resource Information System
KES: Kenya Efficiency Study
KMHFL: Kenya Master Health Facility List
LMIC: Low-Middle Income Country
MOH: Ministry of Health
P and P: Permanent and Pensionable
PFM: Public Finance Management
PHC: Primary Health Care
rhris: Regulatory Human Resource Information System
SCHMT: Sub-county Health Management Team
SDG: Sustainable Development Goals
SERU: Scientific Ethics Review Unit
UHC: Universal Health Coverage
